# An evaluation model for aboveground biomass based on hyperspectral data from field and TM8 in Khorchin grassland, China

**DOI:** 10.1371/journal.pone.0223934

**Published:** 2020-02-28

**Authors:** Xiaohua Zhang, Xiuli Chen, Meirong Tian, Yongjun Fan, Jianjun Ma, Danlu Xing

**Affiliations:** 1 School of Applied Meteorology, Nanjing University of Information Science & Technology, Nanjing, China; 2 Nanjing Institute of Environmental Science, Ministry of Ecology and Environment of China, Nanjing, China; 3 Baotou Teachers’ College, Biological Science and Technology Institute, Baotou, Inner Mongolia, China; 4 College of Life Science, Lang Fang Normal University, Lang Fang, China; Guangzhou University, CHINA

## Abstract

Biomass is an important indicator for monitoring vegetation degradation and productivity. This study tests the applicability of Hyperspectral Remote-Sensing in situ measurements for high-precision estimation aboveground biomass (AGB) on regional scales of Khorchin grassland in Inner Mongolia, China. In order to improve prediction accuracy of AGB which is frequently used as an indicator of aboveground net primary productivity (ANPP), this paper combined ground measurement with remote sensing inversion to build the spectral model. The ground normalized difference vegetation index (SOC_NDVI) calculated from ground spectral of grassland vegetation which was measured by a portable visible/NIR hyperspectral spectrometer (SOC 710). Meanwhile, the remote normalized difference vegetation index (TM_NDVI) calculated from remote spectral of grassland vegetation which was measured by Thematic Mapper (TM) from Landsat 8 which launched by National Aeronautics and Space Administration (NASA). According to regression analysis for the relationship between AGB and SOC_NDVI, SOC_NDVI and TM_NDVI, the evaluation model for aboveground biomass was developed (*AGB* = 12.523×*e*^3.370×(0.462×*TM_NDVI*+0.413)^, standard error = 24.74 g m^-2^, *R*^2^ = 0.636, *p* < 0.001). The model accuracy verification results show that the correlation between the measured value and the predicted value of biomass was better with low model standard error. The model could make up for the lack of timeliness and comprehensiveness of conventional ground biomass survey, and provide technical support for high-precision large-area productivity estimation and ecological degradation diagnosis of regional scale grassland.

## Introduction

Grassland biomass is not only the most important indicator for degraded ecosystem but also for grassland productivity. Therefore, how to improve the accuracy of regional grassland biomass estimation has become a research hotspot. With the development of technique, remote sensing data were increasingly used in the regional vegetation survey such as vegetation biomass [1–5], vegetation cover (VC) [6–7], nitrogen content [8], and the leaf area index of vegetation [1, 9] based on the analysis of relationship between vegetation spectral features and vegetation characteristics. The main method of spectral feature extraction was to describe the spectral features by red, yellow and blue (three sides) optical parameters, Red Valley and Green Peak variables, from which the best reflection spectral band which was most closely related to biomass could be found.

The cost of remote sensing survey was lower than that of field survey at regional scale. Therefore, regional vegetation investigation was carried out by establishing models based on remote sensing data [10–12]. Many vegetation indices which calculated from remote sensing spectral data were used as predictors of parameters, such as the normalised difference vegetation index (NDVI), ratio vegetation index, vegetation condition index, perpendicular vegetation index, soil adjusted vegetation index, and transformed soil adjusted vegetation index [13–17]. NDVI can reduce the interference of solar zenith angle and atmospheric noise, reduce the influence of topography and vegetation community and the impact of light changes [18], improve the reflectance contrast of vegetation and soil[9, 19], meanwhile, NDVI has a wide range of vegetation monitoring and high detection sensitivity [3, 4], therefore, NDVI is widely applied in many studies about vegetation monitoring which can provide possibility for comparative analysis with other results [3, 4, 19–23]. NDVI has been calculated by various satellite data sets such as MODIS, SPOT, TM et al [24–27]. TM 8 satellite data had been chosen in this study, because the narrowband indices of TM 8 were highly suitable to map AGB accurately.

Spectral reflectance especially can be influenced by variable factors of the landscape such as the distribution of plant communities [28], soil colour [29], hydrology[30], and topography[31], and sensor radiance may be strongly affected by atmospheric scattering[32], so it is important to combine the ground hyperspectral survey in the method and develop the model from the perspective of relationship between remote-sensing data and the biophysical properties of vegetation in order to improve the accurate estimation of the grassland aboveground biomass [6,33–37].

Khorchin grassland is one of the four great grasslands in China, it is important to provide the scientific and accurate estimation of AGB for the sustainable utilization of grassland resources. In this study, we aimed to develop a model for estimating AGB of grassland during the growing season in order to improve the estimation accuracy of AGB. We used remote sensing of Landsat 8 and ground hyperspectral to calculate the NDVI, and on-field AGB measured in the same period to establish a model to assess spatial AGB of the Khorchin grassland.

The main objectives of this study are: (i) developed the model of AGB based on Hyperspectral Data from field and TM8 to clarify the corresponding relationship between remote sensing image and the measured vegetation index on the ground of the Khorchin steppe, and (ii) to improve the accuracy in the estimation of AGB calculated by the model, which is related with grass yield, and be able to provide support in guiding the development of the livestock industry in future.

## Materials and methods

### Study area

**Fig 1:**
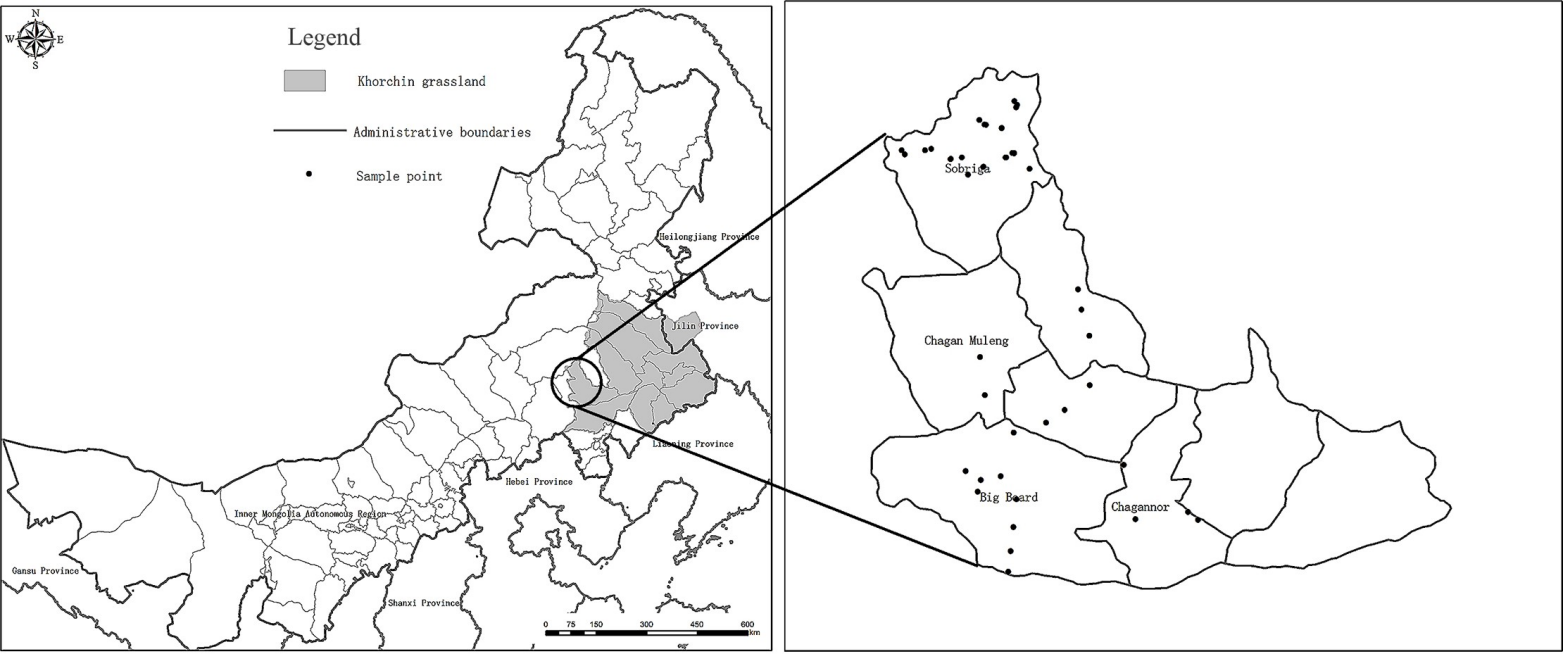
Map of Inner Mongolia (left) and the location of the sampling sites in Bairin Youqi (right).

The Khorchin grassland is one of the four grasslands in China. We choose the region of Bairin Youqi in Inner Mongolia of China which located in the western of Khorchin grassland as the study area (Fig 1), which is an important component of the Khorchin grassland with sensitive and fragile ecology. Bairin Youqi has a semiarid, temperate, continental monsoon climate with mean annual temperature of 4.9°C and mean annual precipitation of 358 mm (precipitation is less than evaporation). The elevation is 400–1900 m above sea level, the main vegetation are meadow, sandy vegetation, and low mountain grassland. The dominant grassland species include *Achnatherum splendens* (*Trin*.) Nevskia, *Stipa capillata* Linn., *Leymus chinensis (Trin*.*)* Tzvel., and *Agropyron cristatum (Linn*.*)* Gaertn.

There were no endangered or protected species in the sturdy area. The research was funded by National Environmental Conservation Research Program; therefore, we achieved the permit of Bairin Youqi government to carry out the research on the state-owned land.

### Field investigation and image process

Field work was conducted during 15–30 July 2016. Based on the topography and land use, 39 plots were established with different types of vegetation. The plot size was set at 30 × 30 m, equivalent to the size of a TM8 pixel. The plots contained a total of 173 quadrats of 1 × 1 m. The data collected were divided into 2 groups. Group one, which contained 153 quadrats were used to build the ground spectral model; group two, which contained 20 quadrats, were used for the accuracy test of the spectral inversion model. Meanwhile, within group one, the data of approximately two thirds of the total quadrats (n = 115) were chosen randomly to build the model while the rest were used for testing the terrain model in terms of selecting the best fitting function and precision.

The ground object spectral were collected using the SOC 710 Hyperspectral Imaging System which Manufactured by Surface Optics Corporation in America. The SOC 710 is a precision instrument with an integrated scanning system and analysis software that can quickly obtain high-quality hyperspectral images at visible to near-infrared (NIR) wavelengths in the range 0.4–1.0 μm. The system can be used under normal lighting conditions at variable exposures and gains. The distance between the SOC sensor and the plant canopy was about 1.2 m, ensuring that the lens is vertically downward, and the diameter of the ground field of view was about 0.2 m.

All hyperspectral image data collected in sunny conditions, in order to reduce the influence of solar irradiance change and the error caused by the instrument itself, the reference plate is measured at the same time in the process of target ground object measurement, and the reflectivity of the reference plate is 1. Meanwhile, the electronic noise of electronic device was affected by electronic system, operating environment, such as temperature, etc., so dark current measurement need to be carried out after the instrument is used for a certain period of time, and the saved results are used for later data processing. One dark current measurement can be carried out for a sample plot. The collected hyperspectral image data were standardized by SRAnal 710 software which belongs to SOC 710 system for reflectivity or radiance calibration.

After the spectral data had been recorded, the standing biomass was collected in the quadrats at each sample location. The fresh weight of standing biomass had been weighed by a balance. Then taken them back to the laboratory and dry them with an oven, record the corresponding spectral data number, dry weight of biomass data and vegetation description of each quadrat.

The satellite data of TM8 were acquired from the Landsat 8 land imager of the United States Geological Survey which imaging time was synchronous with the field investigation time and the images were free of clouds and haze. Four suitable TM8 satellite scenes at PATH/ROWs 123/29, 123/30, 122/29, and 122/30 were analysed. The satellite data were geometrically rectified by Digital Elevation Model (DEM) and GLS2005 [38] ground control points from Land Survey. The four TM8 scenes were processed for atmospheric correction with the Fast Line-of-sight Atmospheric Analysis of Spectral Hypercubes software package.

### Data analysis

We calculated the SOC_NDVI of the samples from SOC 710 spectral reflectance using the ENVI 5.0 image analysis software. The method for calculating NDVI [39] as follows:

$$NDVI=\frac{NIR-RED}{NIR+RED}$$ (1)

Where the RED and NIR bands correspond to wavelengths of 630–680 and 845–885 nm, respectively. Spectral reflectance data should be resampled within the scope of the RED and NIR bands.

The regression analyses were carried out for the scatter diagrams of AGB vs. SOC_NDVI, and SOC_NDVI vs. TM_NDVI. In study area, The 173 quadrats data were employed to obtain the regression model for AGB vs. SOC_NDVI. Mean value of NDVI within a specific plot was calculated, and then the data of the total 39 plots (Fig 1) were used in the regression analysis for SOC_NDVI vs. TM_NDVI.

The coefficient of determination (*R*^2^) and the adjusted *R*^2^ were used to test the relationships between the prediction of NDVI and AGB which measured by satellite data and field data respectively. The standard error (SE, Eq 2) and coefficient of mean error (MEC, Eq 3) were used to test the regression equation accuracy [10].

$$SE=\sqrt{\frac{\sum_{i=1}^{n}{\left(y-y^{\prime }\right)}^{2}}{n}}$$ (2)

$$MEC=\frac{\sum_{i=1}^{n}\left|\frac{y-y^{\prime }}{y}\right|}{n}$$ (3)

Where y is a measured data, y′ is an estimated data, and n is the number of samples.

## Results and discussion

### AGB vs. SOC_NDVI

**Fig 2:**
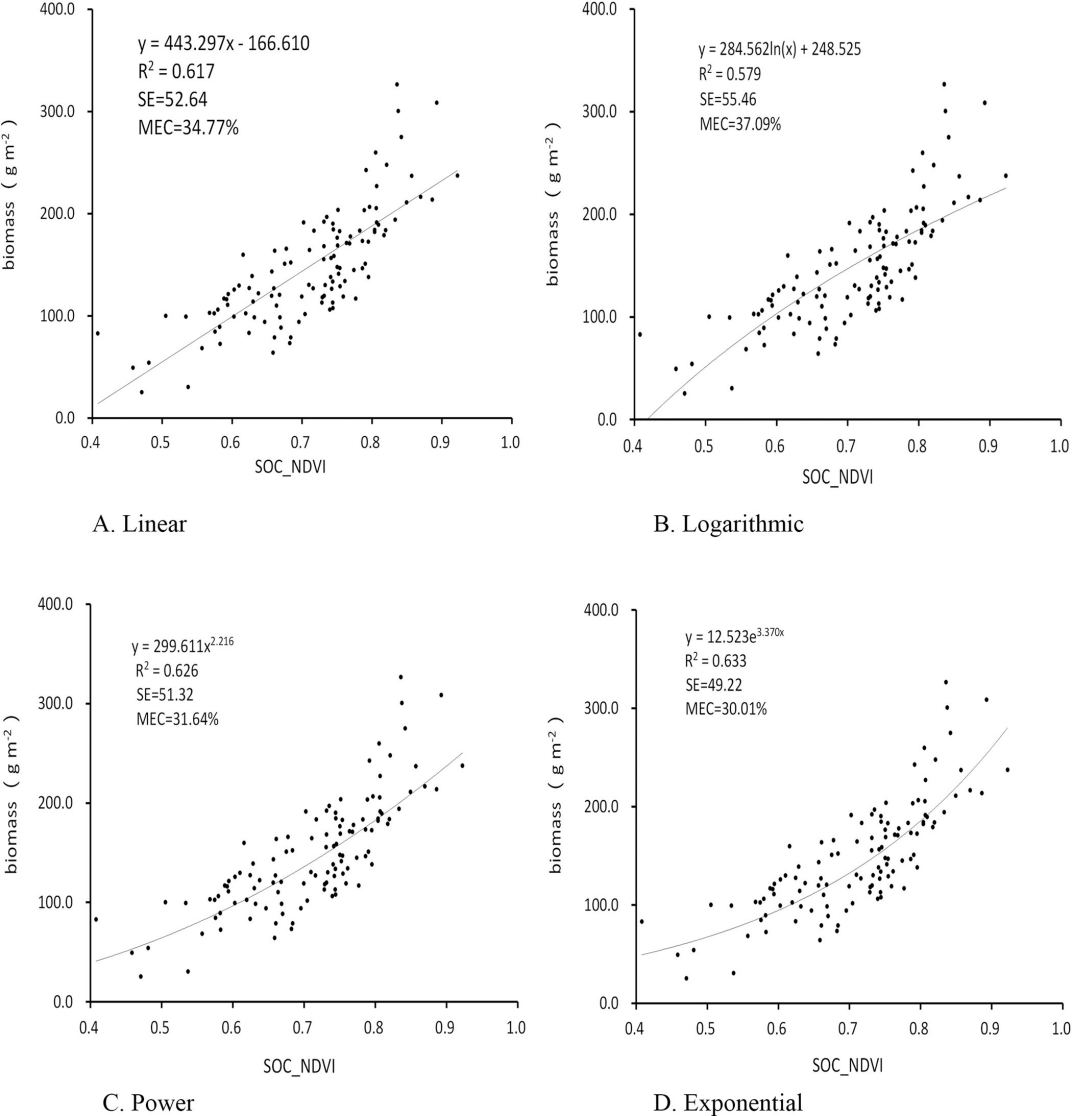
The Simulation curves of the regression equation of the training samples.

The analysis and evaluation of the relevance between AGB and SOC_NDVI which obtained by the SOC 710 in the field was the important step for building the AGB model (Fig 2).

The functions were significant (*P* < 0.001) which consistent with the hypothesis of statistical analysis. The exponential model was superior for AGB, with *R*^2^ of 0.636, indicated by bold type in Table 1.

**Table 1:** Comparison of the regression equations between AGB and SOC_NDVI.

	Linear	Logarithmic	Power	Exponential
*Samples(n)*	115	115	115	115
Equation	*y* = 443.297*x* **−** 166.610	*y* = 284.562ln(*x*) + 248.525	*y* = 299.611*x*^2.216^	***y* = 12.523e**^**3.370x**^
*R*^2^	0.617	0.579	0.626	**0.636**
Adjusted*R*^2^	0.614	0.575	0.623	0.633

The accuracy of the models was tested by SE and MEC (Table 2).

**Table 2:** The errors of the regression equations.

		Linear	Logarithmic	Power	Exponential
AGB	*Samples(n)*	38	38	38	38
	SE (gm^-2^)	52.64	55.46	51.32	**49.22**
	MEC (%)	34.77	37.09	31.64	**30.01**

We choose the most suitable exponential equation for assessing AGB based on *R*^2^ and the independent validations (*R*^2^ = 0.636, SE = 49.22 g m^-2^, MEC = 30.01%; Tables 1 and 2). The relational model of AGB VS. SOC_NDVI of the entire Khorchin grassland as follows:

$$ANPP=12.523\times e^{3.370\times SOC\_NDVI}$$ (4)

### TM_NDVI vs. SOC_NDVI

**Fig 3:**
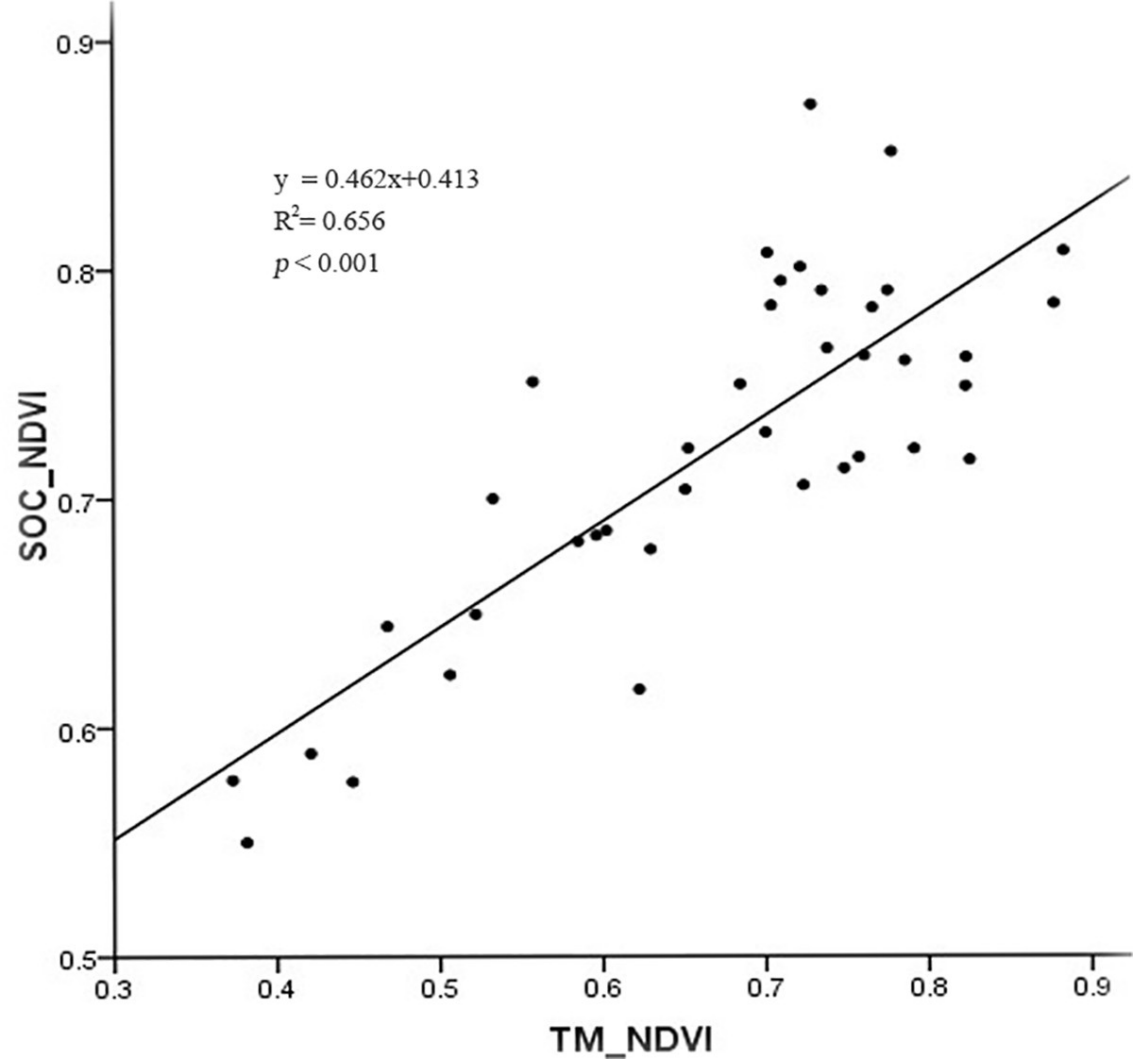
Linear regression between SOC_NDVI and TM_NDVI.

The linear regression equation was selected based on the analysis of the TM_NDVI/SOC_NDVI scatter plot. The relationship between TM_NDVI and SOC_NDVI was significant, with *R*^2^ of 0.656 (*p* < 0.001) (Fig 3). The relational model of TM_NDVI VS. SOC_NDVI for the entire Khorchin grassland as follows (Eq 5):

SOC_NDVI=0.462×TM_NDVI+0.413 (5)

### Spectral inversion model

The spectral inversion model of TM8 for AGB was calculated by Eqs 4 and 5:

$$AGB=12.523\times e^{3.370\times (0.462\times TM\_NDVI+0.413)}$$ (6)

To test the agreement between measured and predicted values, we applied Eq 6 to the TM8 NDVI greyscale image and obtained the patterns of AGB distribution in the study area by grid computing. The test data sets were then converted into vector diagrams defined by geographic coordinates by geographic information system. The values at the test points were recorded in the distribution patterns as the corresponding pixels predicting values of AGB. The relationship between actual and predicted values was used to evaluate the accuracy of model.

**Fig 4:**
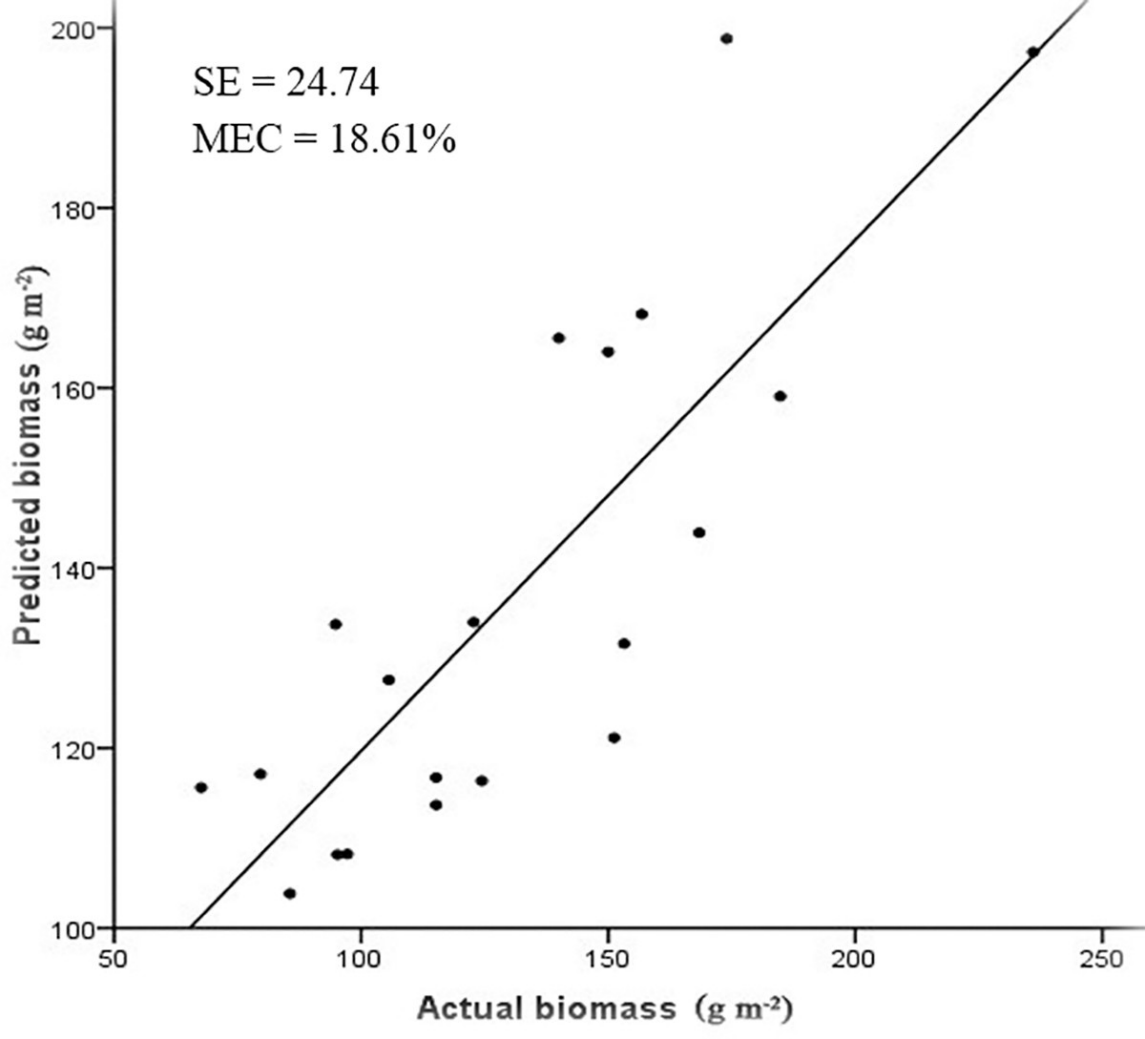
The correlation between predicted biomass and actual biomass.

The correlation between the predicted and actual values of biomass was significant, as were the independent validations for predicting biomass (SE = 24.74, MEC = 18.61%; Fig 4). This study suggested that the spectral inversion model could be used to monitor grassland biomass at regional scales.

### The accuracy of the model

The main goal of this study was to establish the accurate model for estimating AGB of the grassland in Khorchin. The quality of the selected remote sensing image data has a direct impact on the model fitting accuracy [40–42]. In a previous study, we also calculated the NDVI from data collected by a FieldSpec3 spectroradiometer (Analytical Spectral Devices, Boulder, USA) [10]. To ensure that the high-quality data was retrieved to improve the accuracy in the present study, we used the SOC 710 Hyperspectral Imaging System with spectral resolution of 2.3nm which higher than the FieldSpec3 spectroradiometer. Meanwhile, the weight of SOC 710 about half of FieldSpec3 spectroradiometer, so it was more portable for field observation. Compared with the previous Landsat data, Landsat 8 satellite data has been chosen for the higher geometric accuracy and signal-to-noise ratio. In terms of band design, the number of Landsat 8 bands is increasing and the division is more precise, which effectively expands the application range of image data. In terms of imaging mode, the sweep and swing design of the OLI imager has good stability and improves the quality of image acquisition. In terms of geometric accuracy, the L1T data of TM 8 product has been accurately corrected using ground control point GLS2005 and digital elevation model data.

The TM8 remotely sensed imaging data were only released in 2013, so they have not yet been widely applied to monitor vegetational biomass. This study applied the field data for monitoring the vegetation, thereby providing an informational baseline for this study area. The spectral inversion model was ideal, indicating that TM8 remote imaging can be used for research on vegetation biomass on a regional scale.

The optimal equations for the estimation of AGB (Fig 2D) indicate that the correlation of SOC_NDVI VS. AGB from strong to weak at biomass >250 g m^-2^ for grassland. Estimates of biomass above or exceed these levels would inaccurate or unreliable and may be affected by the NDVI lower saturation phenomenon in areas of dense vegetation cover [43].

The result showed that field spectral data collected by SOC 710 are intrinsically linked to those obtained by TM8 remote sensing. However, the model could be more accurate if field and satellite data are collected over several years rather than only for one year. Also, the field and satellite data should be acquired at the same time for maximal correspondence in future field experiments.

## Conclusions

This study developed a relatively accurate model for estimating AGB and tests the applicability of hyperspectral data from field and TM8 to map AGB on regional scales by a regression analysis method. The methodology we adopted in the study was a first attempt to Retrieval of vegetation biomass from ground hyperspectral remote sensing in Khorchin grassland.

The accuracy of ground spectral inversion is affected by many factors, and the quality of the selected remote sensing image data has the greatest impact on the fitting accuracy of the model. Landsat 8 satellite data is selected for remote sensing data, which has higher geometric accuracy and signal-to-noise ratio than previous Landsat data, which effectively expands the application range of image data. In the aspect of imaging mode, the sweep pendulum design of OLI imager has good stability and improves the image quality, and in the aspect of geometric accuracy, L1T data product is a data product after precise correction, and the product accuracy has been greatly improved. In this paper, TM8 data is used to retrieve vegetation biomass, and the results show that calculated *R*^2^ and SE and MEC values for various regression models vary among ground spectral models. By comparison, the best correlation of AGB VS. SOC_NDVI was exponential regression models. An exponential equation was optimal for estimating AGB in the Khorchin grassland. Accuracy verification indicated that the relationship between the actual and predicted biomass was significant. So, it was feasible for Estimating AGB by TM8 satellite data, which accumulates experience for the application of TM8 data in vegetation monitoring field.

The model accuracy could be further improved if spectral monitoring carried out according to vegetation types or more spectral data of ground samples collected. In brief, this research shows the usefulness of hyperspectral data from field and TM8 to evaluate aboveground biomass at very high precision and provide theoretical and data support for RS monitoring, grassland governance and ecological restoration.

## Supporting information

S1 Table The regression analysis data of biomass and SOC_NDVI. (XLSX)

S2 Table The data of TM_NDVI and SOC_NDVI. (XLS)

S3 Table The data of actual biomass and predicted biomass. (XLSX)

S1 Raw Images (PDF)
