# Supplementary material for: An evaluation model for aboveground biomass based on hyperspectral data from field and TM8 in Khorchin grassland, China
Source: PLoS One. 2020 Feb 28;15(2):e0223934. doi: 10.1371/journal.pone.0223934 (PMC7048406; doi:10.1371/journal.pone.0223934)
Supplement: S1 Raw Images — (PDF) [file pone.0223934.s004.pdf]

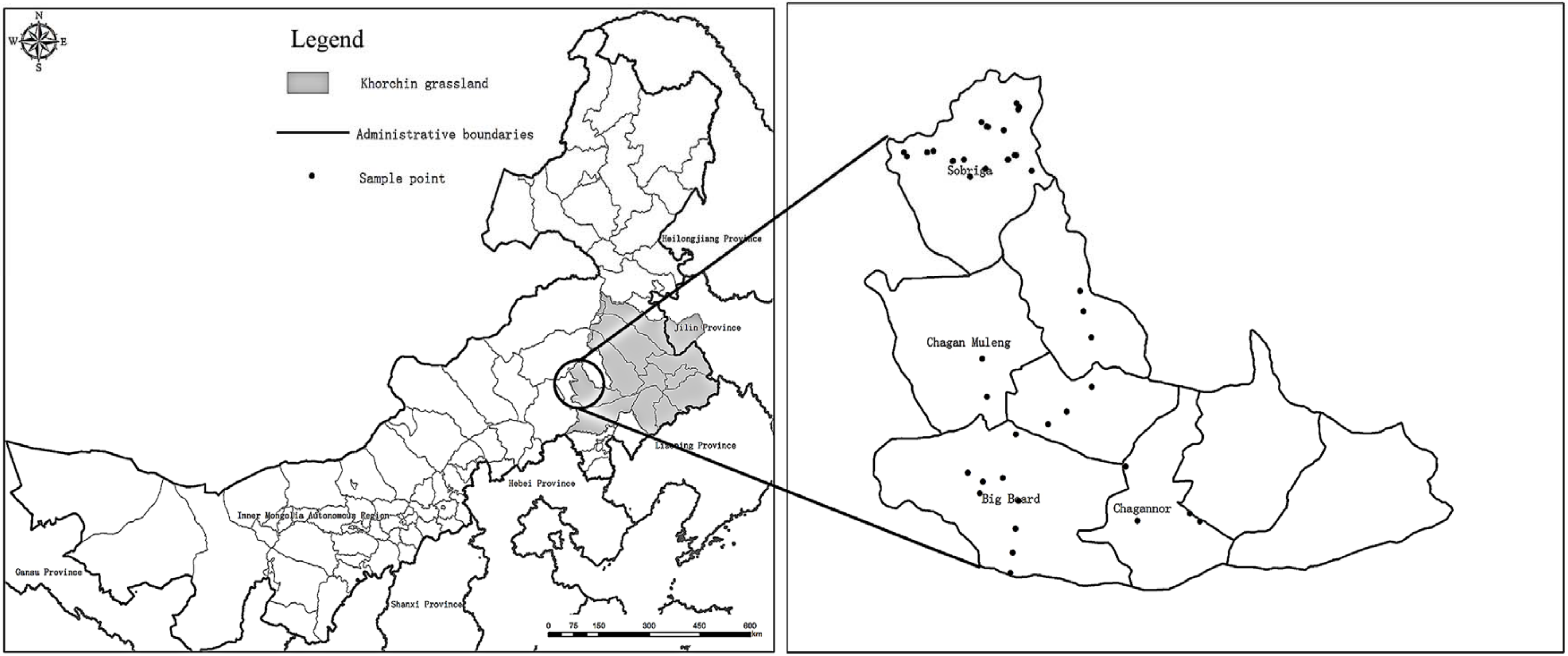

Fig. 1 Map of Inner Mongolia (left) and the location of the sampling sites in Bairin Youqi (right)

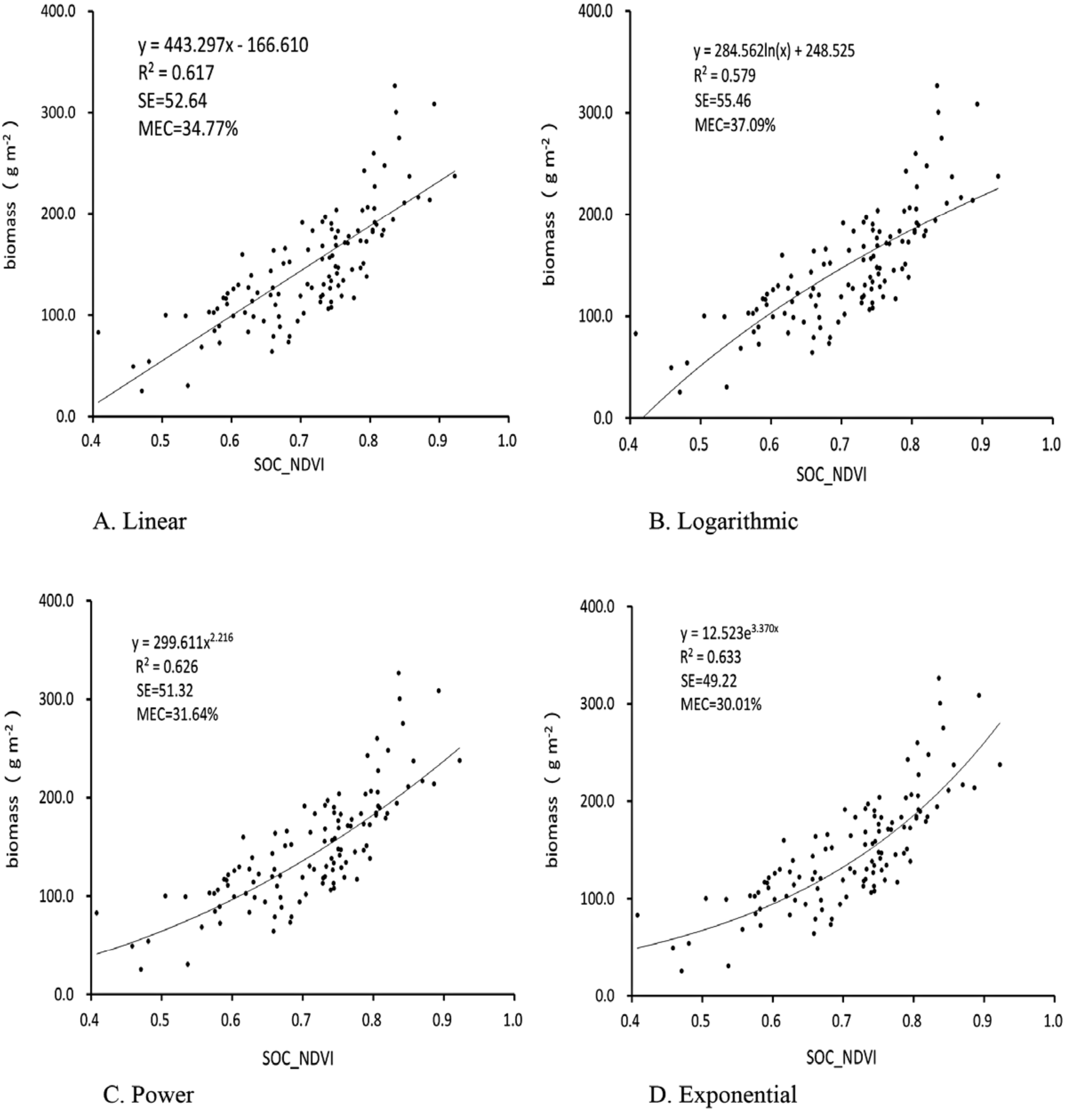

Fig. 2 The Simulation Curves of the Regression Equation of the Training Samples

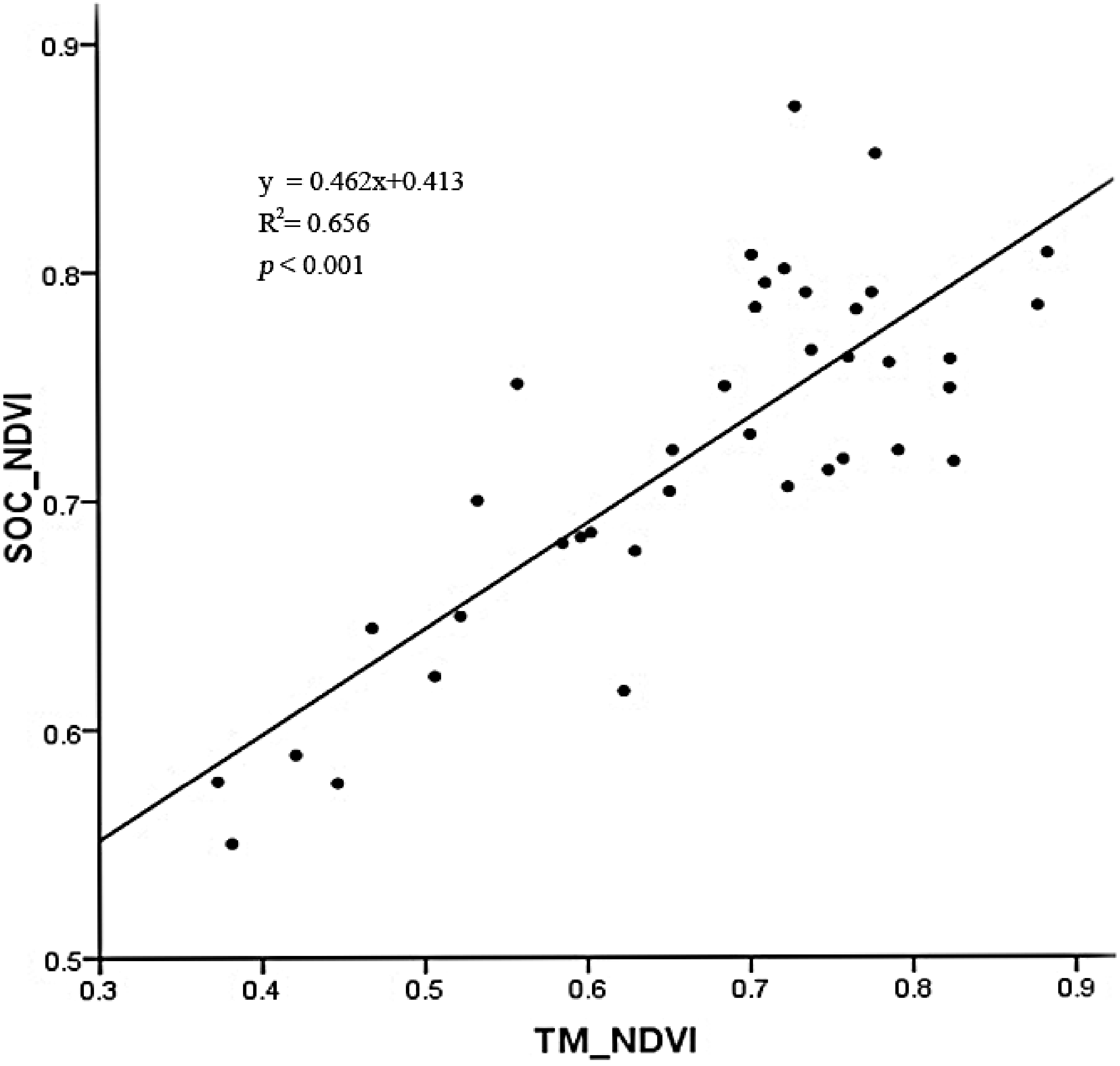

Fig. 3 Linear regression between SOC_NDVI and TM_NDVI

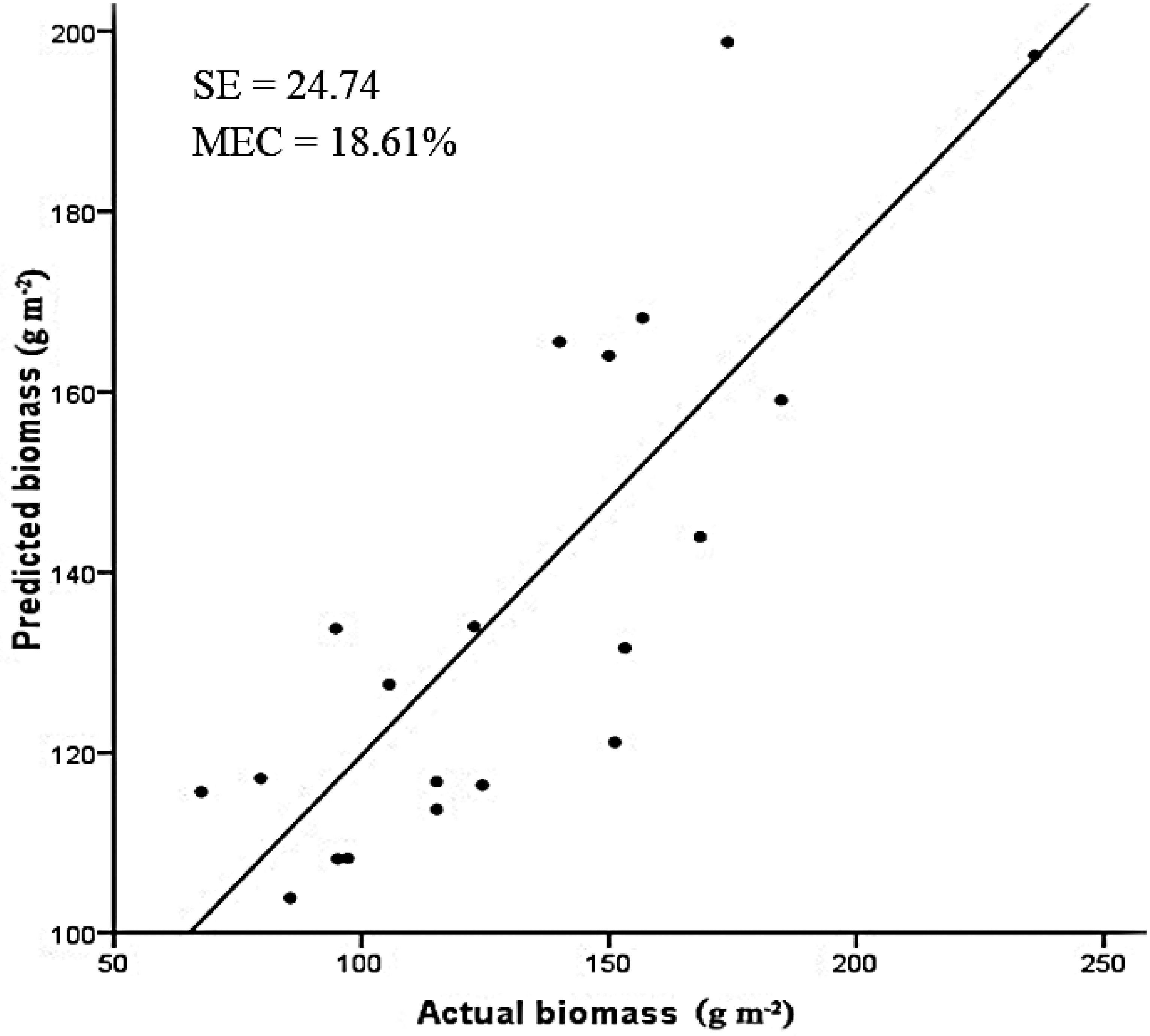

Fig. 4 The correlation between predicted biomass and actual biomass
